# Glycated Hemoglobin and Prediabetes: A Systematic Review of HbA1c Thresholds for Type 2 Diabetes Prevention

**DOI:** 10.3390/jcm15124690

**Published:** 2026-06-17

**Authors:** Dawid Karczewski, Tomasz Karczewski, Mihaela Olsen

**Affiliations:** Cranston Ridge Medical Clinic, Calgary, AB T3M 3A9, Canada; tomasz@cranstonridgemedical.com (T.K.); mihaela@cranstonridgemedical.com (M.O.)

**Keywords:** HbA1c, glycated hemoglobin, prediabetes, type 2 diabetes mellitus, diabetes prevention, diagnostic thresholds, QUADAS-2, GRADE, narrative synthesis

## Abstract

**Background/Objectives**: HbA1c is widely used to identify adults at increased risk of type 2 diabetes mellitus (T2DM), but major guidance differs on whether the lower limit of an HbA1c-defined risk range should be 5.7% (39 mmol/mol) or 6.0% (42 mmol/mol). This systematic review evaluated the prognostic and screening utility of HbA1c 5.7–6.4% compared with HbA1c 6.0–6.4% and examined whether available evidence supports threshold-based allocation of preventive interventions. **Methods**: The review was reported in accordance with PRISMA 2020 and registered in PROSPERO (CRD42019134344). PubMed/MEDLINE, Embase and the Cochrane Library were searched for studies published from 1 January 2016 to 1 January 2026. Eligible evidence comprised human studies in English, French, Hebrew, Italian or Spanish that evaluated HbA1c ranges below the diabetes diagnostic threshold in adults aged at least 40 years or younger adults with established risk factors. Two reviewers independently screened records and extracted data. Risk of bias was assessed using an adapted QUADAS-2 framework for threshold-performance evidence, supplemented by CASP-informed appraisal, and certainty was rated with GRADE domains. Narrative synthesis was selected because populations, thresholds, comparator tests, follow-up and outcome ascertainment were heterogeneous. **Results**: Seven studies were included. Evidence consistently supported a graded risk continuum rather than a single biological cut point. HbA1c 5.7–6.4% identifies more adults but includes many at low short-term absolute risk, whereas HbA1c 6.0–6.4%, especially 6.2–6.4% or combined HbA1c and fasting glucose abnormality, identifies fewer adults at higher near-term risk. Direct evidence comparing 5.7% versus 6.0% thresholds came mainly from one UK cohort, with supportive but indirect evidence from meta-analysis, routine-care cohorts and reversion studies. No trial randomized adults to intervention by HbA1c threshold, and eligible evidence did not directly address early diabetes-related morbidity by threshold. **Conclusions**: HbA1c below the diabetes diagnostic threshold should be interpreted as risk strata, not as a binary disease label. HbA1c 5.7–6.4% is defensible for broad, low-intensity preventive advice, while HbA1c 6.0–6.4% can be used to prioritize structured prevention and closer follow-up. The proposed tiered approach is a pragmatic, hypothesis-generating interpretation of the available evidence rather than a trial-validated intervention algorithm.

## 1. Introduction

Type 2 diabetes mellitus (T2DM) is a major chronic disease burden, and prevention depends on identifying people at sufficiently high risk while glycemic deterioration remains modifiable. HbA1c is central to this strategy because it reflects longer-term glycemic exposure and can be measured without fasting. It is also imperfect: hemoglobin variants, altered erythrocyte turnover, anemia, chronic kidney disease, pregnancy and some ethnicity-related differences can influence interpretation. The threshold used to label an adult as having prediabetes is therefore both a laboratory and a clinical decision, balancing sensitivity, specificity, cost, intervention capacity and patient-centered consequences.

Guidelines differ. The American Diabetes Association defines prediabetes by HbA1c 5.7–6.4% (39–47 mmol/mol), impaired fasting glucose or impaired glucose tolerance [1]. Diabetes Canada defines prediabetes as impaired fasting glucose, impaired glucose tolerance or HbA1c 6.0–6.4% (42–47 mmol/mol) and recommends screening adults aged at least 40 years or at high risk [2,3]. NICE similarly uses HbA1c 42–47 mmol/mol (6.0–6.4%) to indicate high risk and recommends annual testing in people confirmed to be at high risk [4]. The World Health Organization endorsed HbA1c for diagnosing diabetes while emphasizing that HbA1c below the diabetes threshold does not exclude diabetes when glucose-based tests are abnormal [5]. The International Expert Committee recommended HbA1c 6.0–6.4% as a high-risk range, while recognizing that diabetes risk begins below 6.0% [6].

The historical rationale for the 6.0% threshold partly reflects retinopathy risk data, whereas the rationale for the 5.7% threshold reflects prediction of future diabetes at a lower glycemic level [6,7]. Both perspectives are clinically important but answer different questions. A threshold chosen to identify imminent high risk may not be optimal for early prevention; a threshold chosen for broad preventive sensitivity may identify many people whose short-term absolute risk is modest.

Broader identification is not automatically equivalent to clinical benefit. Lowering the threshold can increase sensitivity, but it can also increase overdiagnosis, medicalization, follow-up testing, referral burden and costs, while exposing people to the psychological consequences of being labeled at high risk [8,9]. These concerns are especially relevant in populations with low short-term progression rates, in older adults with competing mortality, or where intervention capacity is limited. A useful HbA1c threshold should therefore be judged not only by how many future diabetes cases it captures, but also by whether the downstream response is proportionate, acceptable and likely to improve patient-centered outcomes.

Preventive interventions can delay or prevent T2DM, particularly among people with combined glycemic and cardiometabolic risk. The Diabetes Prevention Program showed that structured lifestyle intervention and metformin reduced diabetes incidence in high-risk adults, and a Cochrane review confirmed the high risk of progression among people with intermediate hyperglycemia while highlighting heterogeneity across definitions [10,11]. The key question for HbA1c-based screening is therefore not whether prediabetes predicts diabetes, but how far below the diagnostic threshold clinicians should act and how strongly each HbA1c stratum should influence follow-up and intervention intensity.

This systematic review and narrative synthesis evaluates peer-reviewed evidence on HbA1c thresholds for identifying adults at risk of T2DM, with particular attention to the practical comparison between 5.7–6.4% and 6.0–6.4%. The review primarily assesses prognostic utility and screening utility. It separately considers, but does not assume, clinical outcome benefit from allocating interventions by HbA1c threshold because such evidence requires comparative intervention studies rather than threshold-performance studies alone.

## 2. Materials and Methods

### 2.1. Protocol, Registration and Reporting Standard

This systematic review was conducted and reported in accordance with the Preferred Reporting Items for Systematic Reviews and Meta-Analyses (PRISMA) 2020 statement [12]. The completed PRISMA 2020 checklist is provided as Supplementary File S1. A protocol was registered in PROSPERO (CRD42019134344) in 2019. Because the final review was completed after a later evidence update, the registration timeline was explicitly reconciled with the final methods: the core review question, population, index-test thresholds and main outcomes were retained; the search window was updated to include publications through 1 January 2026; and narrative synthesis was used because statistical pooling was not appropriate across heterogeneous designs and outcome definitions. No eligibility changes were made after full-text screening. Early diabetes-related morbidity was retained as a prespecified outcome, but absence of eligible direct evidence was reported explicitly in the Results. During revision, risk-of-bias assessment was strengthened by adding an adapted QUADAS-2 framework, while CASP-informed appraisal and GRADE certainty domains were retained.

The review question was framed as follows: in adults aged at least 40 years, or younger adults with recognized diabetes risk factors, does identification of prediabetes using HbA1c 5.7–6.4% compared with HbA1c 6.0–6.4% better support prevention or delay of T2DM and early diabetes-related morbidity? The question distinguishes three linked but separate issues: prognostic utility, screening utility and evidence for clinical outcome benefit from threshold-based intervention allocation.

### 2.2. Eligibility Criteria

The publication window was 1 January 2016 to 1 January 2026. The 10-year window was selected to capture contemporary HbA1c use while allowing sufficient longitudinal outcome data. Earlier landmark studies were cited only for background where necessary. Eligible publications were human studies in English, French, Hebrew, Italian or Spanish that reported HbA1c-defined prediabetes or HbA1c risk strata below the diabetes diagnostic threshold, and that evaluated progression to T2DM, reversion to normoglycemia, risk discrimination, clinical stratification or early diabetes-related morbidity. The detailed inclusion and exclusion criteria used for study selection are summarized in Table 1.

### 2.3. Information Sources and Search Strategy

PubMed/MEDLINE, Embase and the Cochrane Library were searched. The final searches were executed with coverage through 1 January 2026. Grey-literature and guideline sources were named a priori for threshold context and consisted of the American Diabetes Association, Diabetes Canada, NICE, the World Health Organization and the International Expert Committee. These sources were used to clarify how thresholds are defined and implemented, but the evidence synthesis prioritized peer-reviewed studies. Reference lists of included articles and relevant reviews were checked for additional eligible studies.

The complete database-specific strategies, limits and record-handling details are shown in Table 2. The screening file retained a combined database yield of 41 records before deduplication and 35 records after removal of seven duplicates. Guideline and reference-list checking added one record.

The Cochrane Library strategy was shorter than the Embase syntax because Cochrane uses title/abstract/keyword fields and MeSH descriptors rather than Emtree explosion; the conceptual elements were kept equivalent across databases.

### 2.4. Study Selection and Data Extraction

Two reviewers independently screened titles, abstracts and full texts against the eligibility criteria. Disagreements were resolved by discussion, with a third reviewer available for adjudication. No automated eligibility decisions were used. Extracted data included study design, country, recruitment setting, sample size, age range, baseline HbA1c definition, comparator test, assay or calibration information where reported, follow-up duration, diabetes ascertainment, reversion definition, effect estimates, confidence intervals where available, missing-data handling and limitations relevant to certainty assessment. Outcomes sought were incident T2DM, progression rate, reversion to normoglycemia, predictive performance, positive predictive value, risk discrimination, competing mortality and early diabetes-related clinical outcomes. All eligible results relevant to HbA1c-defined risk strata were considered, irrespective of statistical direction or significance.

### 2.5. Risk of Bias and Certainty Assessment

Risk of bias for primary threshold-performance evidence was assessed using an adapted QUADAS-2 framework because the central question concerns how an index test threshold classifies adults for future-risk stratification [13]. Domains were patient selection, index test, reference standard or outcome ascertainment, and flow and timing. For prognostic cohort studies, the “reference standard” domain was interpreted as the method used to ascertain incident diabetes, reversion, competing mortality or morbidity. Systematic reviews and meta-analyses were appraised using CASP-informed criteria because QUADAS-2 is not designed for reviews [14].

Certainty was rated using GRADE domains: risk of bias, inconsistency, indirectness, imprecision, publication bias and other considerations such as magnitude and consistency of association [15]. Because the evidence base consisted largely of observational cohorts and evidence syntheses rather than randomized threshold-allocation trials, certainty ratings emphasized internal validity, HbA1c calibration, outcome ascertainment, handling of confounding, representativeness and applicability to primary care prevention. Study-level QUADAS-2 and GRADE judgments were arranged from strongest to weakest and are reported below with the certainty findings.

### 2.6. Synthesis

Narrative synthesis was selected because studies differed in population age, ethnicity, baseline risk, index threshold, comparator tests, follow-up duration and outcome definitions. The synthesis prioritized clinically interpretable measures: absolute risk, relative risk or hazard ratio, positive predictive value, reversion to normoglycemia, competing mortality and the proportion of adults classified as high risk by each threshold. Meta-analysis, funnel plots, statistical subgroup analyses and sensitivity analyses were not performed because effect measures, threshold definitions, follow-up periods and outcome ascertainment were not sufficiently comparable for meaningful quantitative pooling.

### 2.7. Reporting Bias Assessment

Risk of bias due to missing results was assessed qualitatively rather than statistically. The number and heterogeneity of included studies precluded funnel-plot analysis or formal small-study-effect testing. To reduce the risk of missing relevant evidence, reference lists of included studies and relevant reviews were checked, guideline and health-agency sources were searched for threshold context, and reasons for full-text exclusion were categorized in the PRISMA flow diagram. Possible publication bias and selective availability of threshold analyses were considered in the certainty assessment.

## 3. Results

### 3.1. Study Selection

**Figure 1:**
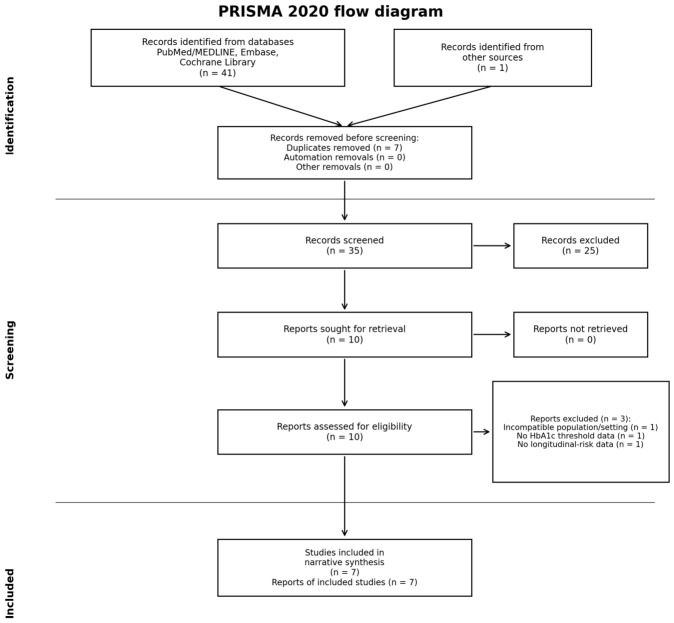
PRISMA 2020 flow diagram for identification, screening and inclusion of studies in the systematic review.

The database searches identified 41 records, and one additional record was identified through other sources. After removal of seven duplicates, 35 records were screened by title and abstract. Twenty-five records were excluded at title/abstract screening, and 10 full-text reports were assessed for eligibility. Three full-text reports were excluded because they did not meet the population, HbA1c-threshold or longitudinal-risk eligibility requirements: one had an incompatible population or setting, one lacked HbA1c-threshold or risk-stratum data, and one lacked longitudinal-risk outcome data. Seven studies were included in the narrative synthesis. The full study-selection process is summarized in Figure 1.

### 3.2. Included Study Characteristics and Mapping to the Review Question

The seven included studies comprised an individual-participant-data meta-analysis, national routine-care laboratory data, prospective community or primary-care cohorts and systematic reviews. Most evidence mapped directly to adults aged at least 40 years or older adults in primary-care or community settings. Evidence for younger adults with risk factors was more indirect: the PREDAPS cohort included adults aged 30–74 years with prediabetes, and broader evidence syntheses included adult populations with biochemical risk states, but no eligible study was designed specifically around younger high-risk adults. No randomized trial was identified that allocated adults to screening or intervention solely according to HbA1c 5.7% versus 6.0% threshold strategies. Included study characteristics and quantitative findings are summarized in Table 3 and Table 4.

### 3.3. Synthesis of Threshold Evidence

The evidence base supports three consistent observations. First, diabetes risk rises continuously across HbA1c values below the diagnostic threshold rather than at a single natural cut point. Second, lowering the threshold from 6.0% to 5.7% markedly increases the number of adults classified as at risk, improving sensitivity but reducing average short-term absolute risk and positive predictive value. Third, adults with HbA1c 6.0–6.4%, especially those also meeting fasting glucose criteria or reaching HbA1c 6.2–6.4%, have higher near-term diabetes risk and lower likelihood of spontaneous reversion.

The direct numerical comparison between HbA1c 5.7% and 6.0% thresholds relied mainly on the Exeter cohort [17]. Other eligible studies examined related but not identical comparisons: Lee et al. compared widely used definitions across cohorts [16]; PREDAPS studies examined combined fasting glucose and HbA1c subgroups and reversion [18,19]; Rooney et al. assessed HbA1c 5.7–6.4% and impaired fasting glucose in older adults [20]; Nicolaisen et al. studied HbA1c 6.0–6.4% in routine Danish care [21]; and Meads et al. synthesized single and combined biochemical definitions [22]. Therefore, the conclusion that 5.7% is more sensitive and 6.0% is more efficient for prioritization is supported by a convergent evidence pattern, but the exact 5.7% versus 6.0% trade-off is not established by multiple direct comparative cohorts or by randomized intervention trials.

### 3.4. HbA1c 5.7–6.4%: Broad Identification and Prevention Sensitivity

The principal strength of the 5.7–6.4% range is sensitivity. It identifies adults before glycemia reaches the higher-risk 6.0–6.4% stratum and offers an opportunity to reinforce weight management, physical activity, dietary quality, blood pressure control and lipid management. This is most appropriate when the intended response is low risk, low intensity and proportionate, such as lifestyle advice, cardiometabolic risk review and repeat testing when clinically indicated.

However, evidence from large cohorts indicates that the 5.7% threshold substantially increases the proportion of adults classified as high risk, including many whose near-term absolute risk is relatively low. In the Exeter cohort, the 5.7% threshold classified more than half of adults aged at least 40 years as high risk, while the 6.0% threshold classified approximately one fifth, with a materially higher positive predictive value [17]. This trade-off is central: 5.7% is useful for broad prevention, but the evidence does not support using it as a stand-alone criterion for intensive intervention.

### 3.5. HbA1c 6.0–6.4% and 6.2–6.4%: Higher Predictive Value and Prioritization

HbA1c 6.0–6.4% has stronger support as a high-risk stratum. Danish routine-care data show that approximately one in five adults with HbA1c-defined prediabetes at 6.0–6.4% progressed to T2DM within five years, even when death was treated as a competing event [21]. The Exeter cohort similarly showed that moving from 5.7% to 6.0% substantially increased 5-year absolute risk and positive predictive value [17].

The evidence also suggests a practical role for HbA1c 6.2–6.4% when intervention capacity is limited. In the Exeter analysis, raising the threshold from 6.0% to 6.2% reduced the number classified as high risk while increasing positive predictive value [17]. NICE similarly prioritizes the upper part of the 6.0–6.4% band when resources constrain intensive lifestyle programs [4]. This does not invalidate the 5.7% threshold; rather, it supports differentiating broad identification from prioritization.

### 3.6. Reversion, Competing Mortality and Heterogeneity of Prediabetes

Prediabetes is not a uniform disease state. PREDAPS analyses showed that reversion to normoglycemia differs substantially according to whether dysglycemia is defined by fasting glucose, HbA1c or both [18,19]. Adults with only one abnormal glycemic marker may revert more often than those with combined abnormalities. This distinction matters because a lower HbA1c threshold may identify a wider group, but many of these adults may not have persistent dysglycemia on repeat testing.

Older age also modifies interpretation. In the ARIC cohort of older adults, a substantial proportion of participants with HbA1c 5.7–6.4% did not progress to diabetes, and death occurred more commonly than progression in this group [20]. Therefore, prevention strategies in older adults should emphasize individualized absolute risk, functional status, life expectancy and cardiovascular risk reduction rather than automatic escalation based only on a prediabetes label.

### 3.7. Early Diabetes-Related Morbidity

Early diabetes-related morbidity was included in the review question, but eligible studies did not directly report whether using HbA1c 5.7–6.4% rather than 6.0–6.4% reduces early diabetes-related morbidity through threshold-based intervention allocation. Some background evidence outside the main eligibility window links HbA1c-defined dysglycemia with cardiovascular or subclinical risk markers [23,24,25,26,27,28,29], but these data do not establish a clinical outcome benefit from assigning preventive interventions by a 5.7% versus 6.0% threshold. The evidence synthesized in this review therefore supports prognostic risk stratification more strongly than morbidity prevention claims.

### 3.8. Risk of Bias and Certainty of Evidence

Risk-of-bias concerns were generally related to observational design, baseline measurement strategy, possible residual confounding, outcome ascertainment and incomplete comparability of HbA1c thresholds across populations. QUADAS-2 judgments most often raised concerns in patient selection and flow/timing because cohorts differed in testing frequency, follow-up completeness, confirmatory testing and routine-care ascertainment. Applicability concerns were most prominent for ethnicity, age and health-system context. The certainty of evidence was rated as moderate for the direction of association between higher HbA1c strata and diabetes progression, low to moderate for using HbA1c 6.0–6.4% to prioritize higher-risk adults, and very low for claiming clinical outcome benefit from a universal HbA1c threshold because no randomized threshold-allocation trial was identified. Outcome-level certainty is summarized in Table 5, and study-level QUADAS-2/GRADE judgments are summarized in Table 6.

### 3.9. Reporting Biases

Formal statistical assessment of publication bias or small-study effects was not appropriate because the review used narrative synthesis and the included studies differed substantially in design, thresholds, outcomes and follow-up. Reporting bias cannot be excluded, particularly for threshold analyses not designed as primary study outcomes and for studies in which HbA1c subgroup analyses may have remained unpublished. However, the consistency of the direction of association across cohort studies, routine-care data and evidence syntheses supports the conclusion that risk increases continuously across HbA1c values below the diagnostic diabetes threshold.

## 4. Discussion

### 4.1. Principal Interpretation

The principal interpretation is that no single, optimal, universal HbA1c threshold for all prevention purposes can be inferred from the available evidence. HbA1c 5.7–6.4% is best interpreted as a wide early-warning range and is defensible for identifying adults who should be offered low-intensity preventive counseling and cardiometabolic risk assessment. HbA1c 6.0–6.4% is better interpreted as a higher-risk stratum for prioritizing structured diabetes prevention, closer follow-up and, in selected high-risk adults, consideration of pharmacologic prevention consistent with existing guidance.

This distinction separates evidence from interpretation. The evidence directly supports prognosis: higher HbA1c values below 6.5% are associated with higher diabetes incidence, and combined glycemic abnormalities confer greater risk than isolated mild abnormalities. The evidence is weaker for screening-policy benefit and weakest for clinical outcome benefit from threshold-based intervention allocation. The proposed tiered framework should therefore be read as a pragmatic synthesis for clinical prioritization, not as a validated algorithm or a new diagnostic classification.

The distinction between broad identification and prioritization resolves much of the apparent tension between guideline thresholds. A low threshold maximizes prevention opportunity but risks over-labeling; a high threshold improves efficiency but may exclude adults who could benefit from earlier behavioral intervention. A tiered approach preserves the advantages of both while reducing the risk that a prediabetes label is treated as a uniform disease state.

### 4.2. Clinical and Public-Health Implications

In primary care, HbA1c 5.7–5.9% may reasonably trigger verification of risk factors, repeat testing when clinically indicated, and low-intensity prevention advice that prioritizes weight, diet quality, physical activity, smoking cessation and cardiovascular risk. This range should not automatically imply intensive intervention, especially in older adults, people with limited life expectancy or populations with low short-term progression rates.

HbA1c 6.0–6.4% may reasonably trigger closer follow-up and a more structured prevention offer. When fasting glucose is also abnormal, or HbA1c is 6.2–6.4%, the evidence supports a stronger case for prioritizing intensive lifestyle programs where capacity is limited. Risk communication should emphasize that progression is not inevitable and reversion is possible, particularly when dysglycemia is mild or isolated.

A practical triage model, presented as hypothesis-generating rather than trial-validated guidance, is: (1) HbA1c 5.7–5.9%: low-intensity prevention and periodic reassessment; (2) HbA1c 6.0–6.1%: structured risk-factor modification and annual follow-up; and (3) HbA1c 6.2–6.4% or combined HbA1c and fasting glucose abnormality: intensive lifestyle intervention and closer clinical monitoring. This model should be tailored to ethnicity, age, comorbidity, medication exposure, body mass index, family history, patient preference and local intervention capacity.

### 4.3. Strengths and Limitations

This review had a clearly stated PICO using accessible language, a contemporary publication window, PRISMA 2020 reporting, MeSH/controlled-vocabulary search logic, defined eligibility criteria, independent review roles, QUADAS-2 risk-of-bias assessment, CASP-informed appraisal and GRADE certainty domains. It also explicitly differentiated prognostic utility, screening utility and clinical outcome benefit, which is essential when comparing HbA1c thresholds.

The primary limitation is the evidence base. Eligible studies are observational for the most part, and no trial directly compared clinical outcomes in patients randomized to intervention allocation by HbA1c 5.7% versus 6.0%. The specific numerical comparison of these thresholds depends heavily on the Exeter cohort, although other studies provide indirect support for graded risk stratification. HbA1c itself has biological and analytic limitations, and diabetes outcomes were confirmed variably by repeat HbA1c, fasting glucose, oral glucose tolerance testing, medication initiation or diagnostic codes.

Applicability to diverse populations is a major limitation. The anchor cohorts informing the synthesis were predominantly European or North American, including the Exeter cohort, Danish routine-care data, PREDAPS and ARIC. The relationship between HbA1c and glycemia can differ by ethnicity, and HbA1c interpretation is also affected by anemia, hemoglobinopathies, kidney disease and erythrocyte turnover [30,31]. The five-language restriction may further compound limited ethnic and geographic diversity. Broad primary-care recommendations should therefore be adapted carefully in populations under-represented in the evidence base.

Additional limitations include the small number of directly relevant studies, selective availability of threshold analyses, possible publication bias, heterogeneity preventing meta-analysis, and inability to apply funnel-plot or statistical reporting-bias methods. The GRADE summary of findings therefore emphasizes where evidence is prognostic and where clinical recommendations remain interpretive.

### 4.4. Research Implications

Future studies should compare threshold-based prevention pathways rather than only threshold-based risk estimates. Pragmatic trials or well-designed emulated target trials could evaluate whether adults with HbA1c 5.7–5.9% benefit from structured lifestyle programs compared with low-intensity prevention, and whether adults with HbA1c 6.2–6.4% require different intensity or follow-up frequency from those with HbA1c 6.0–6.1%. Studies should report ethnicity, anemia status, renal function, hemoglobinopathy considerations, repeat testing, fasting glucose or oral glucose tolerance testing, intervention uptake, competing mortality and early diabetes-related morbidity.

## 5. Conclusions

The principal finding of this review is that HbA1c below the diabetes diagnostic threshold functions best as a graded risk marker, not as a single binary disease label. HbA1c 5.7–6.4% is useful for broad preventive identification when the response is low intensity and proportionate, whereas HbA1c 6.0–6.4%, especially 6.2–6.4% or combined HbA1c and fasting glucose abnormality, better identifies adults at higher near-term risk who may warrant structured prevention and closer monitoring. Because no trial has tested intervention allocation by HbA1c 5.7% versus 6.0%, the tiered approach proposed here should be considered a pragmatic, hypothesis-generating framework that combines HbA1c with repeat testing and individualized risk assessment.

## Figures and Tables

**Table 1:** Eligibility criteria for the systematic review.

Domain	Inclusion Criteria	Exclusion Criteria
Population	Adults aged ≥ 40 years, or younger adults with recognized diabetes risk factors such as obesity, hypertension, dyslipidemia, prior gestational diabetes, family history or high-risk ethnicity.	Established diabetes at baseline; pregnancy-only cohorts; pediatric cohorts; cohorts defined primarily by severe comorbidity unrelated to prevention-oriented screening.
Index test	HbA1c measured using methods aligned with IFCC/NGSP-standardized interpretation, reported as percentage and/or mmol/mol.	Studies in settings where HbA1c calibration was unclear or incompatible with international standards.
Thresholds	HbA1c 5.7–6.4%, 6.0–6.4%, 6.2–6.4% or adjacent strata permitting interpretation of these ranges.	Studies reporting only diagnostic diabetes thresholds without prediabetes or risk-range stratification.
Outcomes	Incident T2DM, progression rate, reversion to normoglycemia, positive predictive value, risk discrimination, competing mortality or early diabetes-related clinical outcomes.	Studies limited to cross-sectional prevalence without interpretable risk or outcome data, unless directly relevant to threshold performance.
Design	Randomized trials, cohort studies, routinely collected data studies, individual-participant-data analyses, systematic reviews and meta-analyses.	Narrative opinion pieces, editorials without data, case reports and animal studies.

**Table 2:** Database search strategies and record handling.

Source	Search Strategy, Limits and Record Handling
PubMed/MEDLINE	**Search string:** (“Prediabetic State”[Mesh] OR prediabet*[tiab] OR “intermediate hyperglyc*”[tiab] OR “non-diabetic hyperglyc*”[tiab]) AND (“Glycated Hemoglobin A”[Mesh] OR HbA1c[tiab] OR A1C[tiab] OR “glycated hemoglobin”[tiab] OR “glycated haemoglobin”[tiab]) AND (cutoff[tiab] OR “cut-off”[tiab] OR threshold*[tiab] OR range*[tiab] OR diagnos*[tiab] OR predict*[tiab] OR progression[tiab] OR regression[tiab] OR reversion[tiab]) AND (“Diabetes Mellitus, Type 2”[Mesh] OR “type 2 diabetes”[tiab] OR T2DM[tiab]) **Limits:** Humans; publication dates 1 January 2016 to 1 January 2026; eligible languages English, French, Hebrew, Italian or Spanish applied at screening. **Record handling:** Included in combined database yield of 41 before deduplication.
Embase	**Search string:** (‘prediabetes’/exp OR prediabet*:ti,ab OR ‘intermediate hyperglycemia’:ti,ab OR ‘non diabetic hyperglycemia’:ti,ab) AND (‘hemoglobin a1c’/exp OR hba1c:ti,ab OR a1c:ti,ab OR ‘glycated hemoglobin’:ti,ab OR ‘glycated haemoglobin’:ti,ab) AND (threshold*:ti,ab OR cutoff*:ti,ab OR ‘cut off’:ti,ab OR range*:ti,ab OR diagnos*:ti,ab OR predict*:ti,ab OR progression:ti,ab OR regression:ti,ab OR reversion:ti,ab) AND (‘type 2 diabetes mellitus’/exp OR ‘type 2 diabetes’:ti,ab OR t2dm:ti,ab) **Limits:** [2016–2026]/py AND [humans]/lim; eligible languages applied at screening. **Record handling:** Included in combined database yield of 41 before deduplication.
Cochrane Library	**Search string:** ([mh “Prediabetic State”] OR prediabet*:ti,ab,kw OR “intermediate hyperglyc*”:ti,ab,kw OR “non-diabetic hyperglyc*”:ti,ab,kw) AND ([mh “Glycated Hemoglobin A”] OR HbA1c:ti,ab,kw OR A1C:ti,ab,kw OR “glycated hemoglobin”:ti,ab,kw OR “glycated haemoglobin”:ti,ab,kw) AND (threshold*:ti,ab,kw OR cutoff*:ti,ab,kw OR “cut-off”:ti,ab,kw OR range*:ti,ab,kw OR diagnos*:ti,ab,kw OR predict*:ti,ab,kw OR progression:ti,ab,kw OR regression:ti,ab,kw OR reversion:ti,ab,kw) AND ([mh “Diabetes Mellitus, Type 2”] OR “type 2 diabetes”:ti,ab,kw OR T2DM:ti,ab,kw) **Limits:** Publication dates 2016–2026. Cochrane-specific syntax used title/abstract/keyword fields plus MeSH descriptors rather than Emtree. **Record handling:** Included in combined database yield of 41 before deduplication.
Other sources	**Source description:** Guideline and health-agency materials from the American Diabetes Association, Diabetes Canada, NICE, WHO and the International Expert Committee; reference lists of included articles and relevant reviews. **Limits:** Used for threshold context and citation chasing. **Record handling:** One additional record identified.

**Table 3:** Included study characteristics, PICO mapping and follow-up.

Study	Design, Setting and Population	PICO Mapping	Thresholds, Follow-Up and Outcomes
Lee et al., 2019 [16]	Individual-participant-data meta-analysis; 16 cohort studies; 76,513 participants; 8208 incident diabetes cases.	Adult cohort evidence for biochemical prediabetes definitions; broad but indirect for the specified age ≥ 40 primary-care PICO.	Compared widely used prediabetes definitions for 5-year diabetes prediction; outcome was incident diabetes.
Rodgers et al., 2021 [17]	Prospective Exeter 10,000 cohort, South West England; 4227 adults without diabetes aged ≥ 40 years.	Directly matches the adult ≥ 40 component of the PICO and provides the clearest 5.7% versus 6.0% comparison.	HbA1c ≥ 5.7%, ≥6.0% and 6.2–6.4%; 5-year HbA1c-defined T2DM risk.
Regidor et al., 2021 [18]	PREDAPS prospective primary-care cohort, Spain; adults with prediabetes.	Primary-care prediabetes cohort; supports interpretation of combined FPG/HbA1c risk but not a universal HbA1c-only threshold.	Prediabetes categories based on fasting plasma glucose and HbA1c; 5-year diabetes onset and reversion.
Giráldez-García et al., 2021 [19]	PREDAPS cohort analysis; adults aged 30–74 years with prediabetes.	Partly addresses younger adults with risk factors, but the results are mainly about reversion rather than intervention allocation.	Reversion to normoglycemia by FPG-defined, HbA1c-defined or combined prediabetes; 3-year follow-up.
Rooney et al., 2021 [20]	ARIC community cohort, United States; older adults without diabetes, mean age about 75 years.	Highly relevant to older adults and competing outcomes; less applicable to younger prevention populations.	HbA1c 5.7–6.4%, impaired fasting glucose, either, or both; median follow-up 5.0 years; outcomes included progression, regression and death.
Nicolaisen et al., 2023 [21]	Nationwide Danish routine-care laboratory data; adults with incident HbA1c-defined prediabetes.	Large routine-care adult population; supports 6.0–6.4% risk stratification but lacks a 5.7–5.9% comparator.	HbA1c 42–47 mmol/mol (6.0–6.4%); maximum 5-year follow-up; T2DM and death outcomes.
Meads et al., 2026 [22]	Systematic review and meta-analysis of biochemical prediabetes tests; 40 original adult studies published 2006–2024.	Broad adult evidence for single and combined biochemical definitions; indirect for the exact 5.7% versus 6.0% allocation question.	Single and combined FPG, glucose tolerance and HbA1c definitions; follow-up varied across included studies.

**Table 4:** Main quantitative findings and methodological interpretation.

Study	Main Quantitative Findings	Methodological Interpretation and Limitations
Lee et al., 2019 [16]	Current prediabetes definitions were associated with approximately four- to eight-fold higher diabetes risk; HRs ranged from 3.78 (95% CI 3.11–4.60) to 8.36 (95% CI 4.88–14.33). Discrimination was similar across definitions, with C-statistics 0.79–0.81.	Strong broad evidence for a risk continuum, but not a direct intervention-allocation comparison of HbA1c 5.7% versus 6.0%. Heterogeneity across cohorts, tests and diabetes ascertainment remained important.
Rodgers et al., 2021 [17]	Overall 5-year risk was 4.2% (95% CI 3.6–4.8). HbA1c ≥ 5.7% classified 56% as high risk, with 5-year risk of 7.1% (95% CI 6.1–8.2). HbA1c ≥ 6.0% classified 22%, with 5-year risk of 14.9% (95% CI 12.6–17.2). HbA1c 6.2–6.4% had 5-year risk of 26.4% (95% CI 22.0–30.5).	Most directly relevant evidence for the 5.7% versus 6.0% trade-off. Findings are observational and from one UK cohort; usual-care follow-up and HbA1c-defined outcomes may affect applicability.
Regidor et al., 2021 [18]	Odds against diabetes onset ranged from 29:1 in isolated FPG 100–109 mg/dL to 1:1 in FPG 110–125 mg/dL plus HbA1c 6.0–6.4%. Reversion ranged from 34.5% (95% CI 28.2–40.5) in isolated HbA1c 5.7–5.9% to 6.2% (95% CI 1.4–10.0) in combined high-risk dysglycemia.	Supports heterogeneity within prediabetes and value of combined testing. Not a direct comparison of universal HbA1c-only thresholds.
Giráldez-García et al., 2021 [19]	Reversion was about 31% for isolated FPG-defined prediabetes, about 31% for isolated HbA1c-defined prediabetes and 7.9% for combined abnormalities.	Directly addresses reversion and persistence of dysglycemia. More informative for heterogeneity and reversibility than for choosing one intervention threshold.
Rooney et al., 2021 [20]	Among participants with HbA1c 5.7–6.4%, 97 (9%) progressed to diabetes, 148 (13%) regressed to normoglycemia and 207 (19%) died. Incidence for total diabetes in HbA1c-defined prediabetes was 22.8 per 1000 person-years (95% CI 18.9–27.5), adjusted HR 3.16 (95% CI 2.22–4.48).	Highlights low short-term progression and competing mortality in older adults. Age structure and race/ethnicity composition limit broad extrapolation.
Nicolaisen et al., 2023 [21]	Median age at prediabetes diagnosis was 66.9 years. Five-year cumulative incidence of T2DM was 21.3% (95% CI 21.1–21.5), and 17.5% (95% CI 17.3–17.7) died within five years.	Large routine-care evidence for the 6.0–6.4% range, but no 5.7–5.9% comparator because the study used the 6.0% definition.
Meads et al., 2026 [22]	The highest meta-analytic HR was reported for IFG 6.1–6.9 mmol/L, HR 9.0 (95% CI 4.6–13.5). Descriptive synthesis found the highest annual diabetes incidence for combined IFG 6.1–6.9 mmol/L + impaired glucose tolerance + HbA1c 6.0–6.4%, at 15.2% per year.	Supports combined glycemic testing and higher-risk stratification. Indirect for the exact HbA1c 5.7% versus 6.0% intervention question.

**Table 5:** GRADE summary of findings.

Finding and Certainty	Evidence Base, Rationale and Interpretation
HbA1c risk continuum (moderate).	Consistent cohort, routine-care and evidence-synthesis findings [16,17,18,19,20,21,22]. Association is biologically plausible and consistent; certainty is limited by observational designs, variable outcome ascertainment and residual confounding.
5.7–6.4% broad identification (moderate).	Supported by the Exeter cohort plus IPD meta-analysis and guideline context [16,17]. A lower threshold identifies more adults and captures earlier risk, but clinical benefit depends on a proportionate low-intensity response and avoidance of over-labeling.
6.0–6.4% higher near-term risk (moderate).	Supported by the Exeter cohort, Danish routine-care data and biochemical-test syntheses [17,21,22]. Higher absolute risk and positive predictive value support prioritization, although evidence is not from randomized threshold-allocation trials.
6.2–6.4% combined abnormality (low-moderate).	Supported by Exeter and PREDAPS analyses and Meads et al. [17,18,19,22]. The pattern is consistent, but subgroup thresholds and combined definitions vary. Best interpreted as prioritization evidence, not definitive treatment guidance.
Threshold-allocation outcome benefit (very low).	No eligible randomized trial compared allocation by HbA1c 5.7% versus 6.0%. Available studies estimate prognosis; they do not demonstrate that one threshold strategy improves morbidity or patient-centered outcomes.
Early morbidity by threshold (very low).	No direct eligible evidence was identified. Morbidity outcomes were not adequately reported by threshold, so claims should be limited to risk stratification and prevention prioritization.

**Table 6:** Study-level QUADAS-2 and GRADE assessment arranged from strongest to weakest certainty.

Rank and Study	Study-Level QUADAS-2/GRADE Judgment and Key Limitation
1. Nicolaisen et al., 2023 [21]	Low/some QUADAS-2 concerns; moderate GRADE contribution for HbA1c 6.0–6.4% progression risk. Large nationwide routine-care dataset with standardized HbA1c range and competing-risk handling. Key limitation: clinical testing patterns may influence ascertainment; no 5.7–5.9% comparator; limited ethnic diversity.
2. Rodgers et al., 2021 [17]	Low/some QUADAS-2 concerns; moderate GRADE contribution for the 5.7% versus 6.0% prognostic trade-off. Adults ≥ 40 from a UK cohort with prespecified baseline thresholds. Key limitation: single UK cohort; usual-care follow-up and HbA1c-defined outcomes may influence event detection.
3. Lee et al., 2019 [16]	Some QUADAS-2/applicability concerns; moderate GRADE contribution for association and discrimination. Individual-participant-data meta-analysis across cohorts and biochemical definitions. Key limitation: diabetes ascertainment, testing and follow-up varied; indirect for threshold-based intervention policy.
4. Rooney et al., 2021 [20]	Low/some QUADAS-2 concerns; moderate GRADE contribution for older-adult prognosis and competing outcomes. Community cohort with HbA1c, FPG, regression and mortality outcomes. Key limitation: highly informative for older adults but less applicable to younger high-risk adults; ethnicity and age modify interpretation.
5. Regidor et al., 2021 [18]	Some QUADAS-2 concerns; low-to-moderate GRADE contribution for heterogeneity and combined-testing interpretation. Spanish primary-care prediabetes cohort followed for five years. Key limitation: selected at-risk primary-care population; not a universal HbA1c-only threshold allocation study.
6. Giráldez-García et al., 2021 [19]	Some QUADAS-2 concerns; low GRADE contribution for reversion evidence. Spanish primary-care adults aged 30–74 years with FPG, HbA1c or combined prediabetes. Key limitation: most informative for reversion rather than incident diabetes or intervention allocation.
7. Meads et al., 2026 [22]	Not a QUADAS-2 primary-study dataset; low-to-moderate GRADE contribution as secondary evidence. Systematic review of 40 adult studies with heterogeneous risk-of-bias profiles. Key limitation: supports combined biochemical risk but not exact HbA1c 5.7% versus 6.0% intervention allocation.

## Data Availability

Data sharing is not applicable because this article analyzes published literature and publicly accessible sources cited in the manuscript.

## Supplementary Materials

The following supporting information can be downloaded at: https://www.mdpi.com/article/10.3390/jcm15124690/s1, Supplementary File S1: Completed PRISMA 2020 checklist [12].
